# Association of Warfarin Therapy with *APOE* and *VKORC1* Genes Polymorphism in Iranian Population

**Published:** 2017

**Authors:** Sajad Rafiee, Masoumeh Rajabibazl, Reza Meshkani, Azam Daraei, Mehryar Zargari, Fahimeh Sharafeddin, Zahra Fazeli, Attabak Toffani Milani, Maryam Taherkhani

**Affiliations:** a *Department of Clinical Biochemistry, Faculty of Medicine, Shahid Beheshti University of Medical Sciences, Tehran, Iran. *; b *School of Advanced Technologies in Medicine, Shahid Beheshti University of Medical Sciences, Tehran, Iran.*; c *Department of Clinical Biochemistry, Faculty of Medicine, Tehran University of Medical Sciences, Tehran, Iran. *; d *Department of Clinical Biochemistry, Faculty of Medicine, Mazandaran University of Medical Sciences, Sari, Iran. *; e *Department of Medical Genetics, Faculty of Medicine, Shahid Beheshti University of Medical Sciences, Tehran, Iran. *; f *Cardiovascular Research Center, Modarres Hospital, Shahid Beheshti University of Medical Sciences, Tehran, Iran.*

**Keywords:** Warfarin therapy, *APOE* polymorphisms, *VKORC1*, Iranian population, Cardio vascular disease

## Abstract

Warfarin is a vitamin K antagonist that genetic and non-genetic factors affected on its dose requirement in the patients with cardio vascular disease. The aim of this study was whether the *APOE* and *VKORC1 *polymorphisms influence on warfarin dose requirements in the part of Iranian patients. Blood samples were collected from 86 warfarin-treated patients. After extraction of genomic DNA, the *VKORC1* (rs9923231) and the *APOE* (rs429358 and rs7412) polymorphisms were genotyped by PCR-RFLP technique. We found that the Iranian patients carrying genotypes GA or AA of *VKORC1* polymorphism tended to receive lower dose of warfarin (p = 0.018). Furthermore, the E3/E3 genotype was observed with the frequency more than 60% in the patients with low dose of warfarin. The BMI and weight also showed a positive correlation with warfarin dose. However, it was not statistically significant (p > 0.05). The results of this study may be useful in defining of warfarin dose algorithms for Iranian patients.

## Introduction

Warfarin is the most commonly anticoagulant drug used in Iran and many other countries. This drug was prescribed for the prophylaxis, treatment of venous and arterial thrombosis (1-3). The studies indicated that low variation in warfarin doses can lead to some severe complications in patients (4). Warfarin dose was determined by prothrombin time international normalization ratio test (PT-INR). Patients received warfarin following PT-INR. It has a target therapeutic range between 2 to 3. PT-INR higher than the therapeutic range associated with increase of the bleeding risk. The risk of thrombosis increases when the PT-INR is lower than the therapeutic range (5-7). 

**Table 1:** Primer sequences and restriction fragments

**Polymorphism**	**Primer sequence(5′ to 3′)**	**Enzyme**	**Size of fragment (bp)**
*VKORC1* rs9923231	F: GCCAGCAGGAGAGGGAAATA R: AGTTTGGACTACAGGTGCCT	MspI	GG: 168 + 122 GA: 290 + 168 + 122 AA: 290
*APOE* rs429358, rs7412	F:GCACGGCTGTCCAAGGAGCTGCAGGC R: GGCGCTCGCGGATGGCGCT	HhaI	E3/E3: 91 E3/E4: 91 + 72 E3/E2: 91 + 83 E2/E4: 91 + 83 + 72

**Table 2:** Demographic and clinical characteristics of patients

**Variable**	**Mean ± SD**
Age (years) Weight (kg) BMI (kg/m^2^) INR Dose (mg/day)	61.49 ± 14.74 76.16 ± 15.25 30.11 ± 5.7 2.4 ± 0.69 3.93 ± 1.75

**Table 3:** Correlation analysis of the clinical variables contrasted with dose of warfarin

**Variable**	**R Score**	**p-value**
Age BMI Weight	-0.3404 0.1877 0.1952	0.001 0.087 0.073

Data were presented by Pearson correlation test. p-value was significant in < 0.05.

**Table 4:** Association of warfarin dose with gender.

**Gender**	**Number**	**Mean warfarin dose (mg/day) ± SD**
Female Male	44 42	4.06 ± 2.01 3.8 ± 1.4

**Table 5:** Genotype and allele frequency of the *VKORC1* rs9923231

**Variant**	**Low dose (%)**	**High dose (%)**	**OR (95% CI)**	**p-value**
Genotype				
GG	11 (21.6)	15 (46.9)	-	**-**
GA	35 (68.6)	16 (50.0)	0.33 (0.12-0.89)	0.028
AA	5 (9.8)	1 (3.1)	0.14 (0.01-1.43)	0.099
Allele				
G	56.7%	68.2%	-	
A	43.3%	31.8%	0.61 (0.32-1.16)	0.137
Dominant model				
GG	11 (21.6)	15 (46.9)	-	
GA + AA	40 (78.4)	17 (53.1)	0.31 (0.11-0.81)	0.018

*Data were presented as n (%).* *Low dose < 5 (mg/day), High dose ≥ 5 (mg/day).* *p-value was significant in < 0.05.*

**Table 6:** Association of *VKORC1* rs9923231 genotypes with daily warfarin dose

**Variable**	**GG**	**GA + AA**	**p-value**	**p-value Adjusted**
Age (year) Gender (male/female) BMI (kg/m^2^) Warfarin dose (mg/day)	56.28 ( 13.60 12/14 31.66 ( 5.48 4.85 ( 2.04	63.55 ( 15.13 30/30 29.39 ( 5.71 3.51 ( 1.46	0.035 0.816 0.097 0.001	- - - 0.009

BMI: Body mass index. p-value was significant in < 0.05.

**Table 7:** Genotype and allele frequency of the *APOE* variants after adjustment

**Variant**	**Low dose (%)**	**High dose (%)**	**p-value**
Genotype E3/E2 E3/E3 E3/E4 E2/E4	5 (9.6) 41 (78.8) 5 (9.6) 1 (1.9)	2 (6.2) 21 (65.6) 7 (21.9) 2 (6.2)	0.304

Data were presented as n (%). p-value was adjusted for age, BMI and gender. p-value was significant in < 0.05.

**Table 8:** Association of *APOE* genotypes with physical parameters and daily warfarin dose.

**Variable**	**E3/E2** **+ ** ** E3/E4** **+ ** ** E2/E4**	**E3/E3**	**p-value**	**p-value Adjusted**
Age (year) Gender (male/female) BMI (kg/m^2^) Warfarin dose (mg/day)	60.36 ( 15.78 9/5 28.31 ( 5.97 4.46 ( 2.34	61.49 ( 14.88 34/39 30.0 ( 5.67 3.80 ( 1.63	0.798 0.256 0.237 0.230	- - - 0.130

BMI = Body Mass Index. p-value was significant in < 0.05.

Warfarin is an anticoagulant that acts by inhibiting vitamin K-dependent clotting factors. The CYP2C9 gene encodes one of the main enzymes involved in the metabolism of warfarin. The studies revealed that several variants of CYP2C9 alleles are associated with reduced enzyme activity and lower clearance rates of warfarin (8). Patients carrying at least one copy of CYP2C9 variant showed reduction of warfarin metabolism and required a daily warfarin dose lower than patients homozygous for the wild-type CYP2C9*1 allele (9-10). 

Warfarin inhibits vitamin K epoxide reductase that leads to the regeneration of reduced form of vitamin K (Hydroquinone) from vitamin K epoxide in the vitamin K cycle (11). The enzyme vitamin K epoxide reductase is encoded by the vitamin K epoxide reductase complex, subunit 1, gene (*VKORC1*). It has been demonstrated that different polymorphisms of this gene were associated with coagulation disorders (9); Patients carrying the 1639G > A polymorphism in the promoter region of the *VKORC1* gene were more sensitive to warfarin and required lower doses.

According to the effects of warfarin, the liver storage level of vitamin K can play a role in requirement of warfarin dose (10). Phylloquinone is the main form of vitamin K which is bound to chylomicron remnants within blood circulation, and *APOE* influences on the liver absorption stage of chylomicrons (11). Apolipoprotein E transports lipids through the circulation from the intestine to the liver and removes them by receptor-mediated endocytosis (12-13). Three common isoforms of the *APOE* molecule were encoded by three common variants of the *APOE *gene that were categorized as *E2*, *E3* and *E4*. These variants differ in nucleotide sequence at two sites (14). *E4* isoform has the most clearance of vitamin-K-rich *APOE* from plasma (15). It has been proposed that *APOE* polymor­phisms might influence the uptake of vitamin K into hepatocytes and oral administration of anticoagulant efficacy (16-17). 

In the initiation period of treatment, the patients are at risk of over anticoagulation or bleeding during the first few months (18-19). These side effects are the result of the variation in genotypes that lead to different responses to the warfarin loading dose (20). Also, drug interactions can play a role in enhancing or lowering effect on warfarin dose (21). The current warfarin dosing algorithms do not incorporate all genetic and environmental factors that could affect warfarin dose requirements. Identification of the new factors affecting anticoagulation response could be useful in the prediction of suitable warfarin dose (20). Although the association of *VKORC1 *and *APOE* polymorphisms with warfarin dose has been performed in different populations, the association of these polymorphisms with warfarin dose was not studied in Iranian population yet. 

The aim of the current study was to investigate a number of factors that might have effect on the variability in warfarin dose requirements in Iranian population.

## Experimental

*Patients*

Patients followed up from February 2014 until September 2014 at Loghman hospital and those who received a stable dose of warfarin were selected. Finally, 86 patients were enrolled from the city of Tehran and nearby areas around it (Caucasian).We scheduled patients with stable warfarin dose that had sequentially remained within therapeutic range for at least a period of 3 months. According to the underlying disorders and clinical conditions, INR therapeutic range was considered 2-3.

Patients showed one of the following indications: atrial fibrillation; heart valves diseases; history of cardiac thromboembolism and venous thromboembolism, with or without pulmonary embolism. Their demographics and medical history were documented. The patients followed their routine treatment until end of the study. Patients with simultaneous liver disease, and those whose medications interact with warfarin metabolism, and patients with out of range INRs were excluded from the study. Genomic DNA was extracted by modified salting-out method (22).

*VKORC1 and APOE genotyping*

Genomic DNA was genotyped for *VKORC1 *SNP (rs9923231) and* APOE* SNPs (rs429358 and rs7412) using PCR-RFLP. The polymerase chain reaction (PCR) was carried out in a final volume of 25 µL containing each 0.4 µM of each primers, 0.2 mM deoxynucleoside triphosphate (dNTP), 0.4 to 0.8 µg genomic DNA, 500 mM KCl, 100 mM Tris–HCl (pH 8.4), 1 U Taq polymerase (Bioneer, South Korea) and 1.5 mM MgCl_2_. The amplification reaction was done in the presence of 5% DMSO for *APOE* SNPs. The primer sequences were as described earlier (Table 1) (2, 19).

The PCR conditions consisted of 32 cycles with the following steps: 94°C denaturation for 30 sec, different annealing dependent on the primer (64 °C for* VKORC1 *SNP and 68 °C for* APOE* SNPs) for 30 sec, 72 °C extension for 60 sec. Initial denaturation and final extension were performed as 95 °C for 6 min and 72 °C for 5 min, respectively. 

The PCR product for *VKORC1 *SNP (rs9923231) and *APOE* SNPs (rs429358 and rs7412) were digested with *Msp*I and *Hha*I enzymes (Fermentase), respectively. Then, they were separated by 12% and 18% polyacrylamide gels, respectively and visualized with DNA silver nitrate staining. The fragments that produced after digestion were presented in Table 1.

*Statistical analysis*

The mean (± SD) were calculated for daily doses administered during the 24-day induction phase, INR values and demographic characteristics. The correlation of age, BMI and weight with dose used in warfarin therapy was analyzed by Pearson correlation test. The patients were divided into two groups depending on dose of warfarin therapy: low dose (less than 5 mg/day) and high dose (more than 5 mg/day). The association of warfarin dose with genotypes of rs9923231*, *rs429358 and rs7412 

were also estimated. 

## Results

*Relationship between demographic characteristics and warfarin dose*

In the present study, 86 patients were genotyped for the *VKORC1* (rs9923231) and the *APOE* (rs429358 and rs7412) polymorphisms. Demographic characteristics of patients were presented in Table 2. The Mean ± SD value of warfarin dose was estimated 3.93 ± 1.75 mg/day. Correlation of age, weight and BMI with warfarin dose is shown in Table 3. As observed in Table 3, as age increased warfarin dose reduced (p-value = 0.001). Also there is positive correlation of BMI and weight with warfarin dose but it is not significant in p-value < 0.05. Association of median warfarin daily dose requirements with gender did not show a large significant variation (Table 4).

*Relationship between VKORC1 gene polymorphism and warfarin dose*

Genotype and allele frequency of the *VKORC1* (rs9923231) variant without adjustment for age, gender and BMI was shown in Table 5. The *GA* genotype showed the most frequency (68%) in the patients with low dose of warfarin. Furthermore, the obtained results showed that those with at least one copy of the *VKORC1 A* allele (*VKORC1 AA* or *GA* genotype) required a lower daily dose. Because of the low frequency of *AA* genotype in population, recessive model wasn’t applicable. Association of *VKORC1 *(rs9923231) genotypes and physical characteristics with warfarin dose was presented in Table 6. The data indicated that the patients with *GA* and *AA* genotypes required lower warfarin maintenance doses and the maintenance of dose in these patients was more difficult than the patients with *GG* genotype. The *VKORC1 *polymorphism had a distinct impact on the warfarin dose. Patients with the *VKORC1 A* allele (*VKORC1* *GA* or *AA* genotype) showed significant difference in the mean dose (3.51 (1.46 mg/day) when compared with *VKORC1 GG* patients (4.85 (2.04 mg/day). However, the patients with *VKORC1 G* allele were less affected than those with *VKORC1 A* by the risk of bleeding and their warfarin requirement did not deviate from the standard dose.

*Relationship between APOE gene polymorphism and warfarin dose*

The frequency of *APOE* genotypes (rs429358 and rs7412) were showed in Table 7. The *E3/E3* was the most frequent genotype in the Iranian population and this genotype was more frequent than other genotypes in low dose category of the patients. Association of *APOE* genotype with clinical parameters and warfarin dose were presented in Table 8. The *E3/E4*, *E3/E2* and *E2/E4* genotypes were not separately analyzed due to low frequency. As observed in Table 8, the association of *APOE* genotypes with warfarin dose was not significant in p-value 0 < 0.05.

## Discussion

Warfarin is one of the most prescribed oral anticoagulants worldwide, and its dose requirements influenced by both environmental and genetic factors (23). Warfarin acts through inhibiting the vitamin K epoxide reductase, which reduced vitamin K in the liver (16). The reduction of vitamin K is essential for the activation of blood clotting factors (21). The uptake of vitamin K into the liver is mediated by apolipoprotein E (12-13). In the present study, the impact of the two most frequent *APOE* polymorphisms was investigated on the quality of warfarin therapy among Iranian patients. 

The *APOE E4 *allele was less common among Caucasians (3), which have limited us to detect the effect of *APOE* in our population. The previous studies showed that the *E4* allele was associated with a higher dose of warfarin in African Americans. Although the *APOE* gene polymorphisms may have effect on warfarin dose in Caucasians, the effect was not clear and a statistically significant effect has less published (3).

Hepatic uptake of chylomicrons, and the vitamin K which is carried by chylomicrons, depends on *APOE* genotypes. *E4* carriers had the most rapid clearance. Thus, the *APOE E4* variant facilitates vitamin K uptake and increases the gamma-carboxylation of vitamin K-dependent clotting factors (16). A contrasting hypothesis is that increasing hepatic clearance of vitamin K is mediated by the *APOE E4 *variant and this variant increased vitamin K catabolism and reduced the availability of coagulation factors to vitamin K (15, 24). An *in-vivo* study has shown that the apolipoprotein *E4* isomer was catabolized twice faster than the *E3* isomer (25). Other investigations have shown that patients carrying the *APOE E4 *allele had a faster lipoproteins uptake by the liver. Their vitamin K levels in blood circulation were also lower than the patients with no *E4* alleles (12, 15, 26). Thus, it seems that patients carrying *E4* alleles had a higher uptake of vitamin K. As showed in Table 8, there was an association between E3/E3 genotype and low dose of warfarin but the genotypes with one E4 allele was accompanied with a higher dose of warfarin. Our study supported the result of former studies.

Kohnke *et al. *(16) have studies on the effects of *APOE* polymorphism on warfarin dose requirement. They have found that the homozygote patients for CYP2C9 wild-type and carrying the* APOE E4/E4* genotype required significantly higher warfarin doses. Although our study had a few patients with* E4/E4* genotype, it was consistent with the obtained results. However, the results obtained from some studies did not show any association between *APOE* genotype and warfarin dose requirement (21).

Although the *E4/E4* genotype was not common and its correlation with lower doses was not clear in this study, we suggested that *APOE* gene polymorphisms may have role or interact with other factors to maintain warfarin doses.

Some other studies indicated that *VKORC1* polymorphisms could influence on warfarin pharmacokinetics as well as *APOE* polymorphisms (9). The allelic variants *VKORC1* *GA* and *AA* increased warfarin half-life and were associated with higher risk of bleeding, exceeded from upper limit of therapeutic INR levels or difficulty in estimating an adequate warfarin dose maintenance (27). Most studies suggested that carriers of *VKORC1* *GA* or *AA* genotypes required lower warfarin doses as compared to wild type individuals to maintain adequate levels of INR.

*VKORC1* effects were discovered in 2004 (9, 28). It encodes an enzyme that regenerates the reduced form of vitamin K, which is responsible for the gamma-carboxylation of vitamin K-dependent clotting factors II (prothrombin), VII, IX, and X in post-translational modifications (29). Warfarin inhibits the *VKORC1* reductase activity (30). Rieder *et al.* (31) reported that the patients who used warfarin could be divided into two low or high dose groups according to their *VKORC1* genotypes.

The other study (32) indicated that *VKORC1* genotyping was useful for prediction the individual variability of warfarin dose, as it accounts for up to a two-fold decrease in warfarin daily requirement among patients within the same age range. Furthermore, Limdi *et al.* (33) have shown that variant *VKORC1* 1173C/T genotype did not increase the risk for major or minor hemorrhage. The results suggested that the analysis of *VKORC1* polymorphisms could increase our understanding from its predictive value in optimizing warfarin therapy. Our results recommended that *VKORC1* genotyping could be helpful in anticoagulant therapy. The obtained results indicated that low dose of warfarin in initiation of therapy for patients with the *VKORC1* variants (*GA *and* AA*) could be accompanied with better treatment.

In summary, our study demonstrates that *APOE* and *VKORC1 *genotypes affected on warfarin maintenance dose requirements in Iranian patients. The results indicated that the genotypes GA or AA of *VKORC1* polymorphism was associated with lower dose of warfarin in Iranian patients. Furthermore, the carriers of E4 alleles need higher dose of warfarin in the treatment phase.
